# Letter to the editor in response to: achievement emotions of medical students: do they predict self-regulated learning and burnout in an online learning environment?

**DOI:** 10.1080/10872981.2023.2259163

**Published:** 2023-09-20

**Authors:** Ava Khoshnaghsh, Mahnoor Irfan, Jamila Tukur Jido, Syeda Anum Zahra

**Affiliations:** a King’s College London, Department of Life Sciences and Medicine, London, United Kingdom; b Department of Medicine, St George’s University of London, London, United Kingdom; c Department of Medicine, University of Leeds, Leeds, United Kingdom; d Imperial College London, Department of Medicine, London, United Kingdom

**Keywords:** Achievement emotions, learning-related anxiety, learning-related boredom, self-regulation, medical student burnout

## Abstract

This letter summarises the perspectives of medical students in the United Kingdom (UK) on the research paper conducted by Wang and colleagues (2023) titled ‘Achievement Emotions of Medical Students: Do They Predict Self-Regulated Learning and Burnout in an Online Learning Environment?’. Overall, we find this paper a positive contribution to the literature surrounding medical education. However, we have highlighted some weaknesses in the study design and proposed additional points for exploration in further iterations of this study. We hope the readership takes the time to read our letter and consider the points we have raised.

## Dear Editor,

We thank you for the publication of the article titled ‘Achievement Emotions of Medical Students: Do they predict self-regulated learning and burnout in an online learning environment?’ [1] As members of the medical community and advocates for advancements in medical education, we wish to share our perspectives. The transition from in-person to online education has gained significant interest, particularly after COVID-19. This article highlights external influences impacting student engagement in online medical education.

The article mentions negative emotions hindering learning strategies. However, the evidence quoted for this from Ahmed et al [2] and Obergriesser et al [3] involves Grade 7 and Grade 4 students, respectively. Stronger evidence is necessary, ideally from studies involving undergraduate medical students. The disparity in student numbers (82 undergraduates, 200 postgraduates) limits drawing a well-founded conclusion due to cohort underrepresentation. This factor should be considered during analysis. Postgraduate students were primarily enrolled in the clinical oncology specialisation; this finding was not explored during data analysis. Oncology professionals may face higher burnout levels [4], linked to the field’s challenging nature, potentially affecting postgraduate emotional burnout.

We have identified potential improvements for future study iterations to enhance their relevance within medical education literature. Several limitations may undermine the study’s representativeness. As highlighted by the authors, the study’s geographical limitations, focusing on a single medical school and only two East Asian countries – Hong Kong and China – introduce selection bias. To address this, we recommend considering an international collaboration in future work, facilitating a more diverse and comprehensive study. Additionally, the small sample size and poor response rate (33.4%, 282 out of 844) raise concerns about potential type 1 error. To mitigate the low response rate, analysing non-response bias and adjusting data collecting for increased validity is crucial. Only 38 students completed the survey’s demographic section, raising representation concerns. Assuming data as missing at random risks bias. Disregarding incomplete responses without proving insignificance could overlook meaningful associations, weakening study validity. Even with missing random data, exclusion may lose valuable information for a comprehensive and accurate analysis.

Data collected solely during the COVID-19 pandemic raises questions about the broader applicability of the findings; considering factors like personal circumstances, mental health, workload, age and gender could influence outcomes. Additionally, using a survey in the study introduces the risk of under- or overreporting of burnout.

We found value in discussing this paper and offer insightful suggestions to readers. Encouraging students to maintain a ‘reflections diary’ could prove beneficial. Furthermore, conducting interviews and mental health sessions between online lectures could provide deeper insights into students’ intentions, motivations and learning goals. Finally, considering the physical burnout symptoms is important. This question can be included in interviews or identified in reflection diaries. Incorporating quantitative and qualitative data to illustrate the pandemic’s global impact, such as analysing interview transcripts, focus group discussions and reflection diaries, can identify recurring patterns related to achievement emotions, self-regulated learning, and burnout.

As the educational landscape evolves, students’ emotional well-being remains paramount when designing effective online medical education.
